# Trait anxiety and unsafe behavior among miners with moderated mediation effects

**DOI:** 10.1038/s41598-026-37390-5

**Published:** 2026-05-06

**Authors:** Wenliang Xia, Wensheng Wang

**Affiliations:** School of Management, China University of Mining and Technology-Beijing, Beijing, 100083 China

**Keywords:** Job anxiety, Trait anxiety, Unsafe behaviors, Unsafe psychological states, Perceived stress, Human behaviour, Psychology and behaviour, Sustainability

## Abstract

This study aims to examine the associations among trait anxiety, job anxiety, unsafe psychological states, perceived stress, and unsafe behaviors among miners, with a particular focus on the moderating role of job anxiety and the mediating roles of unsafe psychological states and perceived stress. A questionnaire survey was conducted among 267 miners to collect data on trait anxiety, job anxiety, unsafe psychological states, perceived stress, and unsafe behaviors. Data were analyzed using the PROCESS macro in SPSS. The results indicate that the association between trait anxiety and unsafe behaviors varies as a function of job anxiety, and that unsafe psychological states and perceived stress are statistically significant mediators in the relationship between trait anxiety and unsafe behaviors. These findings suggest that psychological factors are closely associated with unsafe behaviors among miners and highlight the potential importance of mental health–related considerations in safety management practices within the mining industry. This study provides theoretical support and practical implications for incorporating psychological perspectives into occupational safety management.

## Introduction

In high-risk industries such as coal mining, unsafe behavior remains a central concern in occupational safety management^1^. As traditional safety management systems have gradually improved, academic research has increasingly shifted its attention from observable operational risks to the underlying psychological factors associated with unsafe behavior^2^. Among various emotional traits, trait anxiety has received growing attention as an important psychological characteristic that may be associated with individuals’ tendencies toward unsafe behavior^3^. However, systematic empirical research focusing specifically on coal miners remains relatively limited.

Two key theoretical and empirical gaps can be identified in the existing literature. First, prior studies have predominantly conceptualized anxiety as a transient, state-based emotional response, emphasizing immediate reactions to stressful situations while paying comparatively less attention to stable emotional traits at the personality level^4^. This tendency constrains a comprehensive understanding of the relationship between trait anxiety and unsafe behavior. Existing evidence suggests that individuals with higher levels of trait anxiety are more likely to exhibit persistent unsafe psychological tendencies, which may be associated with biased risk perception and reduced compliance with safety regulations, thereby corresponding to a higher likelihood of rule-related deviations^5^.

Second, the psychological processes underlying the association between trait anxiety and unsafe behavior remain insufficiently clarified. Preliminary studies indicate that unsafe psychological states—such as risk neglect, resistance to safety regulations, and diminished safety awareness—may be statistically associated with unsafe behavior and may function as intermediary psychological conditions^6^. Nevertheless, empirical evidence supporting these relationships remains limited. In addition, work-related situational factors, including heavy workloads and high task intensity, are commonly associated with elevated job anxiety, which may alter the strength of the relationship between trait anxiety and behavioral outcomes^7^. The potential moderating role of job anxiety within this association warrants further empirical examination.

To address these gaps, the present study, drawing on Affective Events Theory and Behavioral Safety Theory, develops a moderated mediation framework to examine the relationships among trait anxiety, unsafe psychological states, perceived stress, job anxiety, and unsafe behavior. Specifically, trait anxiety is examined as an antecedent psychological characteristic associated with unsafe behavior, unsafe psychological states and perceived stress are considered as potential mediating variables, and job anxiety is incorporated as a contextual moderator. This framework emphasizes the interaction between individual traits and situational factors, reflecting a conceptual shift from a purely situation-focused perspective to a trait–situation interaction perspective.

An empirical investigation was conducted among front-line coal miners. Owing to their hazardous working environments, high task demands, and frequent exposure to emotionally salient events, coal miners represent a suitable population for examining associations between emotional traits and unsafe behavior. Compared with workers in lower-risk occupations, miners tend to experience more pronounced psychological stress responses, enhancing the theoretical and practical relevance of this research context.

This study contributes to the existing literature in three main ways. First, it extends current research by systematically examining trait anxiety as a relatively stable psychological characteristic associated with unsafe behavior in high-risk industries. Second, by incorporating unsafe psychological states and perceived stress into a dual mediation framework, it provides a more nuanced understanding of the psychological conditions linked to the association between trait anxiety and unsafe behavior. Third, by introducing job anxiety as a moderating variable, this study explores the contextual boundary conditions under which these associations may vary, thereby extending the application of behavioral safety theory to complex occupational settings. These contributions offer theoretical insights and practical implications for incorporating psychological perspectives into safety management practices.

## Theoretical basis and conceptual model of anxiety’s impact on unsafe behaviors of coal miners

### The relationship between trait anxiety and unsafe behavior

Trait anxiety refers to an individual’s tendency to consistently exhibit emotional responses such as tension, worry, and vigilance when faced with uncertain or potentially threatening situations^8^. It is considered a relatively stable emotional characteristic within one’s personality. Unlike state anxiety, trait anxiety is not triggered by specific events but persists across various times and situations^9^. It is characterized by heightened sensitivity to potential risks, negative expectations, and diminished coping capacity. In high-risk occupational environments, such as coal mining sites, employees with higher levels of trait anxiety are more likely to develop unsafe anticipations during task execution, which may be associated with differences in their behavioral judgment and response selection^10^.

Unsafe behavior refers to actions in which employees violate safety regulations, ignore risk warnings, or engage in unauthorized operations, potentially leading to accidents or increased operational hazards^11^. While some unsafe behaviors may be driven by task-related intentions or experiential knowledge, extensive research has shown that such behaviors are often closely associated with an individual’s emotional state^12^. Negative emotions such as anxiety have been found to be associated with interference in attention, information processing, and behavioral control, which may correspond to a reduced ability to recognize and respond to hazardous situations^13^.

According to the Conservation of Resources Theory, individuals tend to conserve core resources—such as energy and cognitive capacity—when facing resource depletion^14^. Under high trait anxiety, individuals may need to invest continuous effort in emotional monitoring and regulation to maintain internal psychological balance, which may limit the allocation of cognitive resources and attention in the workplace^15^. From a theoretical perspective, such a state of perceived resource scarcity may be associated with reduced sensitivity to external environmental cues and lower levels of compliance with safety norms, thereby corresponding to a higher likelihood of unsafe behavior. Moreover, individuals with high trait anxiety are more prone to catastrophic thinking, which may be linked to excessive reactions to uncertainty and unexpected events in the workplace, potentially resulting in judgment errors or behavioral deviations^16^.

Therefore, this study posits that in coal mining contexts, employees with higher levels of trait anxiety are more likely to engage in unsafe behavior. Based on this reasoning, the following hypothesis is proposed:

- H1: Trait anxiety is positively associated with unsafe behavior among coal miners.

### The mediating relationship between unsafe psychology and stress perception

Unsafe psychological states refer to individuals’ negative mental tendencies—such as a lack of safety perception, distrust in management, and resistance to regulations—when facing operational risks and institutional constraints^17^. Research has shown that individuals with high levels of trait anxiety are more likely to amplify potential dangers during high-risk tasks and develop persistent concerns about their work environment, which may be associated with the emergence of unsafe psychological states^18^. These individuals often experience reduced vigilance and impaired judgment, which may make them more likely to ignore safety instructions and, thereby corresponding to a higher probability of engaging in unsafe behavior.

According to the emotion processing mechanism, emotional states may be associated with behavioral choices by altering individuals’ risk perception structures^19^. As a cognitive mediator, unsafe psychological states make miners with high trait anxiety more prone to avoidance-based responses in hazardous situations, which may intensify their tendency toward rule violations. This mindset may also lead to resistance toward safety regulations, potentially reducing the consistency of compliance behaviors and corresponding to higher levels of unsafe behavior in high-risk work environments. The following hypothesis is proposed:

- Hypothesis H2: Trait anxiety is positively associated with unsafe psychological states among miners.
- Hypothesis H4: Unsafe psychological states are positively associated with unsafe behavior among miners.

Perceived stress refers to individuals’ subjective evaluation of external stressors such as workload, time pressure, and environmental complexity^20^. It reflects the interplay between psychological resource mobilization and perceived coping capacity. In high-risk environments such as coal mines, individuals with high trait anxiety tend to be more sensitive to stimuli, which may make them more prone to heightened tension and perceived resource depletion^21^. As perceived stress increases, cognitive resources may become strained, which may be associated with reduced safety responsiveness and a greater tendency to adopt riskier coping strategies^22^.

Based on the Stress-Appraisal Model, excessive perceived stress may amplify anxiety responses, which may be associated with biased judgment and difficulties in stress regulation^23^. Consequently, miners may be more prone to operational errors or procedural violations. Individuals experiencing high levels of perceived stress may find it difficult to maintain vigilance due to resource depletion or may adopt avoidant coping strategies under psychological defense mechanisms, thereby potentially compromising the quality of their safety-related behaviors^24^. The following hypothesis is proposed:

- Hypothesis H3: Trait anxiety is positively associated with perceived stress among miners.
- Hypothesis H5: Perceived stress is positively associated with unsafe behavior among miners.

In exploring the dual mediation mechanism, existing research suggests that unsafe cognition and perceived stress are not entirely independent, but instead may interact significantly^25^. Specifically, unsafe cognition, driven by negative expectations of the external environment, may be associated with heightened vigilance and sensitivity to potential threats, thereby intensifying individuals’ perception of stress cues in the workplace. In turn, sustained perceived stress may be associated with further depletion of cognitive resources, making individuals more prone to distrust safety mechanisms and react emotionally to operational risks, which may reinforce unsafe cognition.

When facing uncertainty and high task demands, individuals with high levels of trait anxiety are more likely to be simultaneously affected by unsafe cognition and perceived stress, which may be associated with impairments in cognition, judgment, and behavioral regulation, corresponding to a higher likelihood of unsafe behaviors^26^. The dynamic interaction between these two psychological factors not only helps explain the indirect associations between anxiety and behavior but also provides theoretical support for the proposed dual mediation model, highlighting the psychological plausibility and contextual adaptability of the path from emotional traits to risky behaviors. The following hypothesis is proposed:

- Hypothesis H6: Unsafe psychological states and perceived stress mediate the relationship between trait anxiety and unsafe behavior.

### The moderating effect of job anxiety

Job anxiety refers to a sustained state of tension experienced by individuals when facing specific job demands and responsibilities, often arising from concerns about inadequate abilities, limited resources, or potential negative consequences of failure^27^. Unlike trait anxiety, job anxiety is more situational in nature and is influenced by multiple factors such as task intensity, organizational support, and the overall work climate^28^. For miners working in high-risk positions, job anxiety often manifests as persistent worry about potential accidents, heightened sensitivity to failure, and psychological pressure stemming from role overload^29^.

According to Conservation of Resources (COR) theory, individuals in high-anxiety states tend to reduce energy investment and avoid challenging tasks as a means of conserving internal resources and minimizing risk^30^. While this “energy-saving” strategy may serve as a protective mechanism, it may also be associated with weaker adherence to safety regulations, corresponding to a higher likelihood of unsafe behavior. Moreover, job anxiety may heighten individuals’ sensitivity to threat-related cues and reinforce negative perceptions of the organizational system, potentially amplifying the association between emotional states and behavioral responses.

Existing research suggests that the influence of emotional variables on behavior is often moderated by context-specific emotional factors^31^. As a stable individual difference, trait anxiety does not exert a uniform association with behavior; rather, its relationship with behavioral outcomes may vary depending on the intensity of anxiety experienced within specific job contexts^32^. Under conditions of high job anxiety, the latent risks associated with trait anxiety may be more salient, making individuals more likely to adopt unsafe behaviors as a coping response, thereby reflecting a potential “magnification effect” in risk-related actions^33^. Therefore, the following hypothesis is proposed:

- H7: Job anxiety positively moderates the relationship between trait anxiety and unsafe behavior.

### Conceptual model construction

**Fig. 1:**
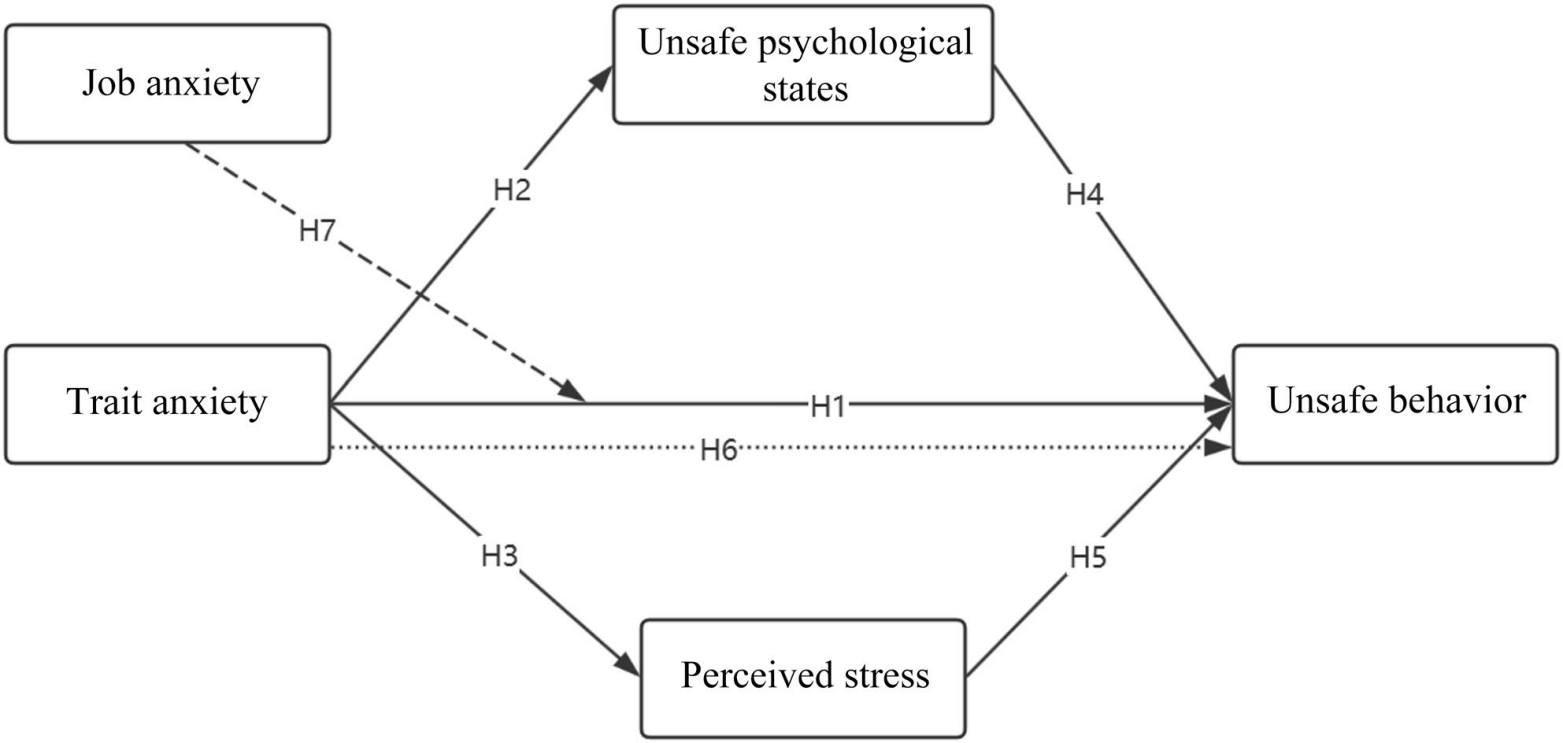
Conceptual model.

Based on the above hypotheses, the constructed conceptual model is shown in Fig. 1. The figure illustrates the relationships between job anxiety, trait anxiety, and unsafe behavior, highlighting the roles of unsafe psychology and stress perception in this process. Through various hypothesized paths (H1-H7), the model explores the associations among psychological factors and individual unsafe behavior, reflecting the potential role of anxiety-related variables in behavioral safety within the work environment. This analysis provides a theoretical foundation for understanding the interaction between psychological states and behavior.

## Measurement

### Sample and procedure

To enhance the scientific rigor and representativeness of the study, front-line coal miners from four major coal-producing regions in China—Shandong, Henan, Inner Mongolia, and Xinjiang—were selected as research participants. These regions are rich in coal resources and have a high concentration of mining enterprises. Miners in these areas are typically exposed to intense physical workloads and elevated safety risks, making them highly representative of the broader coal mining workforce. The sample covered various geographical regions and mine types, which contributes to improving the external validity of the research findings.

A structured questionnaire was used to collect data. The questionnaire consisted of two parts: measurement scales for key variables and basic demographic information, ensuring data completeness and reliability for subsequent analysis. The survey was conducted between October and December 2024, coinciding with a peak period in coal production, to increase the accuracy and authenticity of participants’ recall and responses regarding their work conditions. The data collection process adhered to the principles of voluntariness, anonymity, and confidentiality. Prior to participation, the research team explained the purpose of the study to all respondents and obtained informed consent.

A total of 320 questionnaires were distributed. After excluding those with significant missing data or deemed invalid, 267 valid responses were retained, resulting in an effective response rate of 83.44%. The sample size meets the basic requirements for structural equation modeling. Descriptive statistics of the valid sample’s demographic characteristics are as follows: 98.5% of the respondents were male, and 1.5% were female; 50.2% were aged 31–45, followed by 27.7% aged 46–60, and 22.1% aged 18–30. Regarding educational background, 43.8% held a high school or vocational qualification; 46.4% had more than 10 years of coal mining experience; 78.3% were married; and 98.1% self-reported being in good health, with only 1.9% reporting occupational illness or physical disability. Detailed demographic characteristics are presented in Table 1.

Overall, the sample shows a well-distributed representation in terms of age, education level, work experience, marital status, and health condition, accurately reflecting the demographic profile of front-line coal miners in China.

**Table 1:** Description of the distribution of sample Characteristics.

Item	Description	Frequency	Percentage
Age	18–30 years	59	22.1%
	31–45 years	134	50.2%
	46–60 years	74	27.7%
Education	Middle school and below	63	23.6%
	High school and vocational	117	43.8%
	Associate degree	75	28.1%
	Bachelor’s degree	11	4.1%
	Master’s degree and above	1	0.4%
Work Experience	Less than 1 year	19	7.1%
	1–5 years	62	23.2%
	5–10 years	62	23.2%
	More than 10 years	124	46.4%
Marital Status	Single	47	17.6%
	Married	209	78.3%
	Divorced	11	4.1%
Health Status	Healthy	262	98.1%
	Occupational disease or disability	5	1.9%

### Measurement instruments

(1) Trait Anxiety Scale

This study used the Trait Anxiety subscale of the State–Trait Anxiety Inventory (STAI), developed by Charles D. Spielberger and colleagues, to measure individuals’ levels of trait anxiety^34^. The scale contains multiple items, with sample statements such as “I often feel nervous and restless” and “I tend to worry about small matters.” Participants rated each item based on their actual experiences to reflect their general tendency toward anxiety in daily life. In the present study, the scale demonstrated good internal consistency, with a Cronbach’s alpha coefficient of 0.873.

(2) Job Anxiety Scale

To assess miners’ work-related anxiety levels, this study adopted the Job Anxiety Scale (JAS), a subscale of the Work Stress Scale developed by Parker and DeCotiis^35^. This scale reflects sustained anxiety arising from work demands, lack of resources, or fear of failure. Sample items include “I often worry about not being able to complete my tasks” and “My job makes me feel continuously tense.” Participant responses reflect the degree of anxiety related to their work. The scale has been widely applied in high-risk professions such as mining, healthcare, and education, and is known for its strong reliability and practical value. In this study, the Cronbach’s alpha coefficient was 0.862.

(3) Unsafe Psychological State Scale

Unsafe psychological states were measured using selected items from the Safety Psychology Questionnaire developed by Li Naiwen and Jiang Qiumin^36^. The scale includes four dimensions: resistance to authority, feelings of helplessness regarding safety, decreased safety alertness, and disregard for safety regulations. It systematically captures individuals’ psychological responses to potential risks in high-risk work environments. Participants rated each item based on their own experiences. Sample items include “I can only rely on fate when it comes to possible risks at work” and “After working underground for a long time, I’ve become used to others’ violations of rules.” In this study, the scale demonstrated high internal consistency, with a Cronbach’s alpha coefficient of 0.955.

(4) Perceived Stress Scale (PSS-14)

This study employed the Chinese version of the 14-item Perceived Stress Scale (PSS-14), revised to assess participants’ subjective perception of stress^37^. The scale evaluates the imbalance between environmental demands and coping capacity and consists of two dimensions: perceived tension and perceived lack of control. Sample items include “I was upset because something unexpected happened” and “I felt that I was unable to control the important things in my life.” The scale has been widely used in workplace stress and mental health research, and has demonstrated good reliability and validity. In this study, the Cronbach’s alpha coefficient was 0.828.

(5) Unsafe Behavior Scale

Unsafe behavior was measured using a questionnaire developed by Neal and Griffin during a psychological study of unsafe behavior among coal miners^38^. The scale is specifically designed to assess miners’ unsafe practices during work operations and has been widely applied in coal mine safety research. Sample items include “I occasionally make mistakes because I fail to notice safety warning signs” and “I sometimes use my hands instead of the required tools to complete tasks.” In this study, the scale demonstrated excellent internal consistency, with a Cronbach’s alpha coefficient of 0.958.

### Statistical methods

This study employed SPSS 27 and AMOS 24.0 to conduct a series of data analyses. First, Cronbach’s alpha coefficients were used to assess the internal consistency reliability of the scales measuring trait anxiety, unsafe psychological states, perceived stress, and unsafe behavior. Confirmatory factor analysis (CFA) was then conducted using AMOS, and model fit was evaluated based on indices such as CFI, TLI, and RMSEA to examine convergent and discriminant validity. To control for common method bias, Harman’s single-factor test was applied, and the results indicated that the bias was within an acceptable range. Descriptive statistics, including means, standard deviations, skewness, and kurtosis, were calculated, followed by Pearson correlation analysis. The core hypotheses were tested using the PROCESS macro for SPSS (version 4.0), which was used to analyze both mediation and moderation effects. A bootstrapping procedure with 5,000 resamples was applied to generate 95% confidence intervals, enhancing the robustness of the results. Structural equation modeling was conducted using the maximum likelihood (ML) estimation method, and a small number of missing values were present in the dataset. Missing data were handled using full information maximum likelihood (FIML) in AMOS to improve the accuracy and reliability of model estimation.

### Ethics statement

This study was conducted in accordance with the Declaration of Helsinki and was approved by the Ethics Committee of the School of Management, China University of Mining and Technology, Beijing (Approval No. CUMTB-202408001). All participants were fully informed of the study’s purpose and procedures and provided written informed consent prior to participation. Participation was voluntary, and all data were collected anonymously to ensure confidentiality.

## Results

### Confirmatory factor analysis

In this study, confirmatory factor analysis (CFA) was conducted using AMOS 24.0 to evaluate the discriminant validity of the five-factor model. As shown in Table 2, the results indicated a good model fit: *χ*^2^ (2076) = 3334.829, *χ*^2^/*df* = 1.606, RMSEA = 0.048, CFI = 0.912, and TLI = 0.901. According to commonly accepted fit criteria (RMSEA < 0.08; CFI and TLI > 0.90), the model demonstrated satisfactory fit to the data. Furthermore, the five-factor model outperformed competing models (e.g., four-factor and three-factor models), providing further support for the discriminant validity and structural soundness of the latent variables in this study.

**Table 2:** Results of confirmatory factor analysis (CFA).

Model	χ^2^	df	RMSEA	CFI	TLI
Five-factor model: TA; JA; PS; UC; UB	3334.829	2076	0.048	0.912	0.901
Four-factor model: (TA + JA); PS; UC; UB	7222.120	2271	0.091	0.655	0.644
Three-factor model: (TA + JA); (PS + UC); UB	8062.367	2274	0.098	0.597	0.584
Two-factor model: (TA + JA + PS + UC); UB	8547.279	2276	0.102	0.563	0.549
One-factor model: (TA + JA + PS + UC + UB)	9055.089	2277	0.106	0.528	0.513

TA = Trait Anxiety; JA = Job Anxiety; PS = Perceived Stress; UC = Unsafe Cognition (Unsafe Psychological State); UB = Unsafe Behavior. “+” indicates that factors were merged.

### Common method bias test

This study utilized self-report questionnaires for data collection. Although procedural controls such as anonymous responses and random ordering of items were employed to mitigate the risk of common method bias (Podsakoff et al., 2003), statistical testing was still necessary to ensure the rigor of the conclusions. Accordingly, Harman’s single-factor test was conducted by performing an unrotated exploratory factor analysis on all measurement items. The results showed that the first factor accounted for 35.12% of the total variance, which is below the commonly accepted threshold of 40%, indicating that serious common method bias was not present in this study. Moreover, the major constructs demonstrated good discriminant validity and a reasonable data structure, supporting the adequacy of the data for subsequent structural equation modeling. Therefore, it can be concluded that common method bias was within an acceptable range, and the study’s findings possess satisfactory internal validity and explanatory power.

### **Descriptive statistics analysis**

As shown in Table 3, trait anxiety, job anxiety, unsafe psychological state, perceived stress, and unsafe behavior were all significantly positively correlated. Among them, the correlation between unsafe psychological state and unsafe behavior was the strongest (*r* = 0.768**). Job anxiety also showed strong correlations with unsafe psychological state (*r* = 0.636**) and unsafe behavior (*r* = 0.550**). Trait anxiety was significantly associated with unsafe psychological state (*r* = 0.586**) and unsafe behavior (*r* = 0.554**) and showed a weak but significant positive correlation with perceived stress (*r* = 0.275*). In addition, perceived stress was highly correlated with unsafe behavior (*r* = 0.738**).

Overall, anxiety levels, psychological states, and perceived stress were all significantly associated with unsafe behavior, suggesting that psychological factors are closely related to miners’ safety-related behavior. The skewness (0.121 to 0.991) and kurtosis (− 0.540 to 0.942) of all variables were within acceptable ranges, suggesting that the data were approximately normally distributed and met the assumptions required for subsequent analyses.

**Table 3:** Correlation analysis among key Variables.

Variable	M	SD	Kurtosis	Skewness	1	2	3	4	5
Trait anxiety	48.700	10.837	0.942	0.121	1				
Job anxiety	13.130	5.205	0.508	0.991	0.530**	1			
Unsafe cognition	45.520	18.269	0.336	0.612	0.586**	0.636**	1		
Perceived stress	42.410	7.168	-0.540	0.226	0.275*	0.311**	0.499**	1	
Unsafe behavior	20.080	9.789	0.246	0.662	0.554**	0.550**	0.768**	0.738**	1

*N* = 267; *p* < 0.001***, *p* < 0.01**, *p* < 0.05.

### Hypothesis testing

(1) Main Effects Testing

To test Hypotheses H1 through H5, this study conducted multiple linear regression analyses with unsafe cognition (M1), perceived stress (M2), and unsafe behavior (Y) as dependent variables, trait anxiety (X) as the independent variable, and age, education level, work experience, marital status, and health condition as control variables. The results are presented in Table 4.

In the regression model with unsafe cognition as the dependent variable, trait anxiety showed a significant positive association (β = 0.970, *p* < 0.001), supporting Hypothesis H2. In the perceived stress model, trait anxiety also showed a significant positive association with perceived stress (β = 0.173, *p* < 0.001), supporting Hypothesis H3.

In the model with unsafe behavior as the dependent variable, trait anxiety showed a significant positive association with unsafe behavior (β = 0.134, *p* < 0.001), supporting Hypothesis H1. Moreover, unsafe cognition (β = 0.210, *p* < 0.001) and perceived stress (β = 0.633, *p* < 0.001) were both significantly associated with unsafe behavior, lending support to Hypotheses H4 and H5, respectively. In addition, health conditions significantly predicted both M1 and M2, indicating that an individual’s health status may be associated with unsafe behavior through psychological pathways.

In summary, trait anxiety was significantly associated with unsafe behavior, and this relationship was also reflected through statistical associations involving unsafe cognition and perceived stress, thereby providing further support for the theoretical model proposed in this study.

**Table 4:** Regression analysis of direct effects.

Variable	M1		M2		Y	
	β	SE	β	SE	β	SE
Age	1.470	1.836	0.135	0.850	0.354	0.577
Education	0.636	1.176	0.179	0.545	-0.621	0.368
Work experience	-0.608	1.156	-0.902	0.535	-0.112	0.363
Marital status	-3.351	2.404	0.063	1.113	-0.861	0.759
Health status	15.097*	6.694	7.600*	3.100	-1.640	2.120
X	0.970***	0.084	0.173***	0.039	0.134***	0.033
W					0.130	0.074
X*W					0.008*	0.004
M1					0.210***	0.024
M2					0.633***	0.047
R^2^	0.362		0.111		0.787	
F	25.564***		5.410***		94.837***	

*N* = 267; ***p < 0.001, **p < 0.01, *p* < 0.05;M1 = Unsafe Cognition, M2 = Perceived Stress, Y = Unsafe Behavior, X = Trait Anxiety, W = Job Anxiety, XW = Interaction Term.

(2) Test of Mediation Effects

To examine whether trait anxiety influences unsafe behavior through two mediating variables—unsafe cognition and perceived stress—this study employed a bootstrap mediation analysis. The number of resamples was set to 5,000, and a 95% confidence interval (*CI*) was constructed to enhance the robustness of the results. The analysis results are shown in Table 5.

In the path from X (trait anxiety) → M1 (unsafe cognition) → Y (unsafe behavior), the direct effect was 0.140 (SE = 0.033, 95% CI = [0.074, 0.205]), and the indirect effect was 0.210 (SE = 0.035, 95% CI = [0.148, 0.286]). In the path from X → M2 (perceived stress) → Y, the direct effect remained at 0.140, and the indirect effect was 0.114 (SE = 0.031, 95% CI = [0.054, 0.173]). Since the confidence intervals for both indirect effects did not include zero, the results indicate that unsafe cognition and perceived stress function as statistically significant mediators in the relationship between trait anxiety and unsafe behavior.

In summary, the dual mediation model is supported, indicating that trait anxiety is indirectly associated with unsafe behavior through unsafe cognition and perceived stress, thereby providing empirical support for Hypothesis H6.

**Table 5:** Mediation effect analysis.

Variable	β	SE	LLCI	ULCI
Path	X→M1→Y			
Direct effect	0.140	0.033	0.074	0.205
Indirect effect	0.210	0.035	0.148	0.286
Path	X→M2→Y			
Direct effect	0.140	0.033	0.074	0.205
Indirect effect	0.114	0.031	0.054	0.173

X = Trait Anxiety, M1 = Unsafe Cognition, M2 = Perceived Stress, Y = Unsafe Behavior.

(3) Test of Moderating Effect

To examine the moderating role of job anxiety in the relationship between trait anxiety and unsafe behavior, an interaction term (Trait Anxiety × Job Anxiety) was introduced into the regression model. The results are presented in Table 4.

Regression analysis showed that trait anxiety was significantly associated with unsafe behavior (β = 0.134, *p* < 0.001), and the interaction term was also significant (β = 0.008, *p* < 0.05), indicating that job anxiety moderates the association between trait anxiety and unsafe behavior. In addition, both unsafe cognition (β = 0.210, *p* < 0.001) and perceived stress (β = 0.633, *p* < 0.001) were significantly associated with unsafe behavior.

**Fig. 2:**
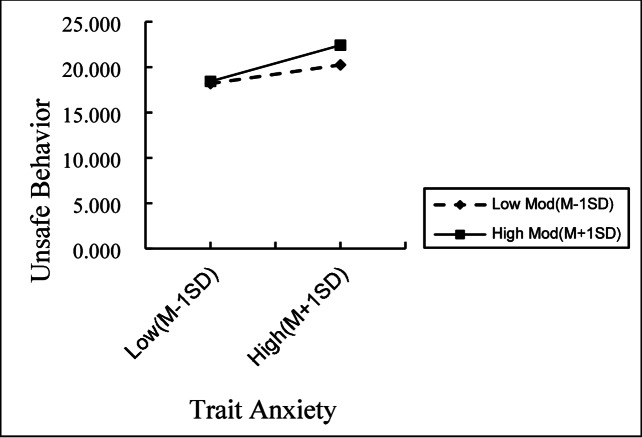
Moderating effect diagram.

Furthermore, as shown in Fig. 2, the slope of the relationship between trait anxiety and unsafe behavior was steeper under high job anxiety conditions (+ 1 SD), suggesting a stronger association between trait anxiety and unsafe behavior when job anxiety was high. In contrast, this association was much weaker under low job anxiety conditions (− 1 SD).

In summary, job anxiety strengthens the association between trait anxiety and unsafe behavior, supporting its moderating role and providing empirical support for Hypothesis H7.

## Discussion

### Discussion of findings and theoretical implications

First, previous research on unsafe behavior has largely focused on external situational factors such as work environment, institutional regulations, and transient emotional states, while relatively little attention has been paid to individuals’ stable internal traits^39^. Using coal miners as the research sample, this study incorporated trait anxiety into the analytical framework, examining its association with unsafe behavior from a personality perspective. The findings extend the theoretical boundaries of traditional safety behavior research by showing that trait anxiety is significantly and positively associated with unsafe behavior, suggesting that long-term emotional tendencies may serve as an important psychological basis for workplace violations^40^.

Second, grounded in the stress-appraisal model and Conservation of Resources (COR) theory, this study examined the mediating roles of unsafe cognition and perceived stress in the relationship between trait anxiety and unsafe behavior. The results showed that individuals with high trait anxiety tend to report heightened sensitivity to risks and a lack of safety in their work, which may be associated with the formation of unsafe cognitive patterns and a higher likelihood of rule-related deviations. Additionally, these individuals may experience greater resource depletion and emotional overload, corresponding to elevated perceived stress, which may be linked to differences in judgment and behavioral responses^41^. These findings enhance our understanding of how emotional characteristics are associated with safety behavior through internal psychological processes, addressing existing gaps in the exploration of emotion–behavior pathways.

Third, this study examined the moderating role of job anxiety in the relationship between trait anxiety and unsafe behavior. The results indicated that higher levels of job anxiety strengthen the association between trait anxiety and unsafe behavior. This finding highlights contextual variability in how personality traits relate to behavioral outcomes and emphasizes the interaction between trait-based and situational emotional factors^42^. It also provides theoretical insight into why individuals with similar levels of trait anxiety may display different behavioral patterns in high-risk occupations.

Fourth, by constructing an integrated “dual mediation and moderation” model, this study systematically illustrated the psychological pathways linking trait anxiety and unsafe behavior. The results supported the mediating roles of unsafe cognition and perceived stress and demonstrated how job anxiety may function as a contextual factor along this pathway. These findings enrich the combined application of COR theory and safety behavior theory and offer theoretical implications for understanding emotion-related safety risks in high-risk industries.

### Practical implications

The findings of this study suggest several practical implications for safety management in high-risk industries, particularly the coal mining sector:

First, managers should pay close attention to the role of stable emotional traits in employees’ safety-related behaviors, especially the association between trait anxiety and unsafe behavior^43^. Companies may consider incorporating emotional health assessments during onboarding or routine evaluations to identify employees with higher levels of trait anxiety. Establishing risk personnel databases and implementing targeted support mechanisms may help address potential safety risks at an early stage.

Second, unsafe cognition and perceived stress appear to function as important psychological pathways linking emotional traits and behavioral outcomes^44^. Organizations may benefit from safety psychology education and emotional management training to help employees better perceive risks and strengthen safety-related beliefs, which may reduce tendencies toward unsafe cognition. In addition, flexible task allocation and workload adjustment mechanisms may help alleviate perceived stress and reduce the likelihood of maladaptive decision-making under sustained psychological strain.

Third, job anxiety was found to strengthen the association between trait anxiety and unsafe behavior. This finding suggests that managers should remain attentive to employees’ emotional fluctuations during periods of high work intensity^45^. During peak production cycles or under conditions of limited organizational support, providing temporary psychological support and humanistic care resources may help mitigate the compounded risks associated with elevated trait and job anxiety.

Fourth, organizations should promote a culture of “psychological safety” at the institutional level, encouraging open and supportive emotional expression to help reduce the accumulation of hidden stressors^46^. This may enhance employees’ trust and sense of belonging. At the same time, companies should establish mechanisms for reporting emotional issues and professional intervention pathways, promoting the institutionalization and procedural integration of psychological care, which may help mitigate the negative cognitive and behavioral responses of highly anxious individuals to risk.

Fifth, organizations may adopt differentiated management strategies based on individual employee characteristics^47^. For example, employees with higher levels of trait anxiety may benefit from personalized counseling resources, flexible working arrangements, or enhanced peer support systems, which may buffer the behavioral manifestations of anxiety and foster a more supportive working environment.

### Limitations and directions for future research

Despite the theoretical and empirical contributions of this study, several limitations should be acknowledged and addressed in future research:

1. Limitations in research design. This study adopted a cross-sectional questionnaire design, which—despite the use of multi-variable control and statistical methods to enhance internal and external validity—cannot establish causal relationships between variables. Future research may consider longitudinal designs or experimental methods, such as multi-wave data collection or intervention-based studies, to dynamically track the relationship between emotional variables and unsafe behaviors and more accurately uncover causal mechanisms.
2. Limited sample representativeness. The sample was drawn from coal miners in selected coal-producing regions of China. While this population has contextual relevance and practical significance, the geographic and occupational concentration of the sample may limit the generalizability of the findings. Future studies should consider broader sampling across various industries and regions, particularly in other high-risk sectors such as construction, electricity, and chemical manufacturing, to improve external validity.
3. Reliance on self-reported data. All variables in this study were measured using self-report questionnaires, which may be subject to social desirability bias, interpretation bias, and other subjective influences. Although Harman’s single-factor test was used to assess and control for common method bias, its effects cannot be completely ruled out. Future research is encouraged to incorporate multi-source data—such as supervisor ratings, peer evaluations, or behavioral observations—to improve objectivity and enable cross-validation.
4. Theoretical model scope. While this study was grounded in the Stress-appraisal model and Conservation of Resources theory and constructed a dual mediation and moderation model, other relevant psychological variables—such as self-efficacy, safety climate, and organizational support—were not included. Future studies could consider incorporating these factors as additional mediators or moderators to further enrich the understanding of how emotional traits influence unsafe behavior.

## Conclusion

Grounded in the Conservation of Resources (COR) theory and the Stress-Appraisal model, this study examined the relationship between trait anxiety and individuals’ unsafe behavior through internal psychological processes. A structural model featuring dual mediators and a moderator was developed and empirically tested to examine the proposed theoretical framework. The results indicate that trait anxiety is significantly associated with miners’ unsafe behavior and that this association is reflected through two psychological pathways: unsafe cognition and perceived stress. These findings help elucidate the internal psychological processes linking emotional traits and behavioral outcomes.

In addition, the study found that job anxiety moderates the association between trait anxiety and unsafe behavior, suggesting that situational emotional states may alter how stable emotional traits relate to behavioral outcomes. This result extends the application of COR theory to complex occupational contexts and underscores the importance of contextual factors in understanding emotion–behavior relationships.

## Data Availability

All data generated or analyzed during this study are included in this published article.
